# The F-Box Protein MEC-15 (FBXW9) Promotes Synaptic Transmission in GABAergic Motor Neurons in *C. elegans*

**DOI:** 10.1371/journal.pone.0059132

**Published:** 2013-03-18

**Authors:** Yu Sun, Zhitao Hu, Yannick Goeb, Lars Dreier

**Affiliations:** 1 Department of Neurobiology, David Geffen School of Medicine, University of California Los Angeles, Los Angeles, California, United States of America; 2 Department of Molecular Biology, Massachusetts General Hospital, Boston, Massachusetts, United States of America; CNRS UMR7275, France

## Abstract

Ubiquitination controls the activity of many proteins and has been implicated in almost every aspect of neuronal cell biology. Characterizing the precise function of ubiquitin ligases, the enzymes that catalyze ubiquitination of target proteins, is key to understanding distinct functions of ubiquitination. F-box proteins are the variable subunits of the large family of SCF ubiquitin ligases and are responsible for binding and recognizing specific ubiquitination targets. Here, we investigated the function of the F-box protein MEC-15 (FBXW9), one of a small number of F-box proteins evolutionarily conserved from *C. elegans* to mammals. *mec-15* is widely expressed in the nervous system including GABAergic and cholinergic motor neurons. Electrophysiological and behavioral analyses indicate that GABAergic synaptic transmission is reduced in *mec-15* mutants while cholinergic transmission appears normal. In the absence of MEC-15, the abundance of the synaptic vesicle protein SNB-1 (synaptobrevin) is reduced at synapses and increased in cell bodies of GABAergic motor neurons, suggesting that MEC-15 affects the trafficking of SNB-1 between cell bodies and synapses and may promote GABA release by regulating the abundance of SNB-1 at synapses.

## Introduction

Ubiquitination is a posttranslational modification that is involved in most aspects of cell biology. It controls the activity of proteins by promoting proteasomal or lysosomal degradation or by modulating the activity of targeted proteins. Many neuron-specific processes are affected by ubiquitination including axon outgrowth, synapse formation and elimination, and synaptic transmission [1], [2]. Ubiquitin ligases catalyze the final step of the ubiquitination reaction. Several hundred predicted ubiquitin ligases are encoded in the genomes of multicellular organisms, and many ubiquitin ligases appear to have a small number of specific target proteins. Determining the function of specific ubiquitin ligases is crucial to understand how ubiquitination controls neuronal function. However, a large fraction of predicted ubiquitin ligases has not yet been studied [3].

SCF ubiquitin ligases are a subfamily of ubiquitin ligases (SCF stands for three subunits, Skp1, cullins, and F-box proteins) [4]. The F-box subunit mediates specificity of ubiquitination by direct interaction with target proteins. The genomes of *C. elegans*, flies and mammals encode about 520, 20 and 100 F-box proteins, respectively [5], [6]. Only eight of these F-box proteins appear to be evolutionarily conserved from *C. elegans* to mammals [6]–[8]. Two of the conserved F-box proteins, FSN-1 (FBXO45 in mammals) and LIN-23 (β-TrCP), have roles in axon outgrowth, synapse formation and regulation of glutamate receptors in *C. elegans* [9]–[14], and homologs of FSN-1 in flies (DFsn) and mammals are involved in axon outgrowth, synapse formation and synaptic transmission [15]–[17]. SEL-10 (FBXW7) functions in the developmental elimination of synapses in *C. elegans* [18], and the mammalian F-box protein Scrapper (FBXL20) ubiquitinates the active zone RIM1 to promote degradation of RIM1 and control neurotransmitter release [19]. Thus, conserved F-box proteins have diverse functions in the nervous system.

In *C. elegans*, mutations in the conserved F-box protein *mec-15* (FBXW9) result in defects in mechanosensation and synapse formation in touch receptor neurons [20], [21]. We further investigated the neuronal functions of MEC-15. *mec-15* is widely expressed in the nervous system, including both cholinergic and GABAergic motor neurons. Using behavioral, electrophysiological and imaging approaches, we found that MEC-15 promotes neurotransmitter release from GABAergic, but not cholinergic, motor neurons, possibly by controlling the abundance of SNB-1 at synapses.

## Results

### Absence of MEC-15 in GABAergic Motor Neurons causes Behavioral Defects

**Figure 1:**
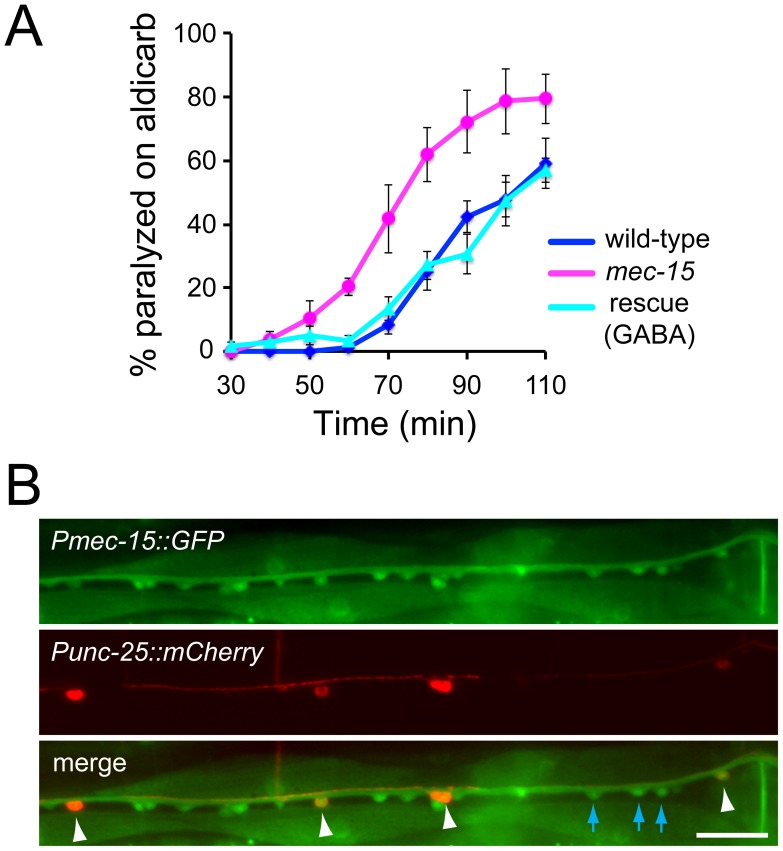
Behavioral defects in *mec-15* mutants and expression of *mec-15.* (**A**) *mec-15* mutants paralyze faster than wild-type control animals in the aldicarb assay. Paralysis of 20 animals of the indicated genotypes was scored over 2 hours. Fast paralysis of *mec-15* mutants is rescued by expressing *mec-15* specifically in GABAergic neurons (rescue). Data are averages from 3 independent experiments +/− standard error of the mean (SEM). (**B**) Representative fluorescence images of young adults expressing GFP under the control of the *mec-15* promoter (*Pmec-15*::GFP, top panel) and mCherry in GABAergic motor neurons (*Punc-25*::mCherry, middle panel). Co-expression of GFP and mCherry indicates expression of *mec-15* in GABAergic motor neurons (cell bodies are labeled with white arrowheads in the merged image). Expression of GFP alone indicates expression of *mec-15* in cholinergic motor neurons (three such cell bodies are labeled with blue arrows). Scale bar is 20 µm.

To study the role of MEC-15 in the *C. elegans* nervous system, we obtained an allele of *mec-15(tm2691)* with a 352 base pair deletion at the N-terminus that is predicted to result in a frame shift and early stop codon and behaves like a complete loss of function of *mec-15* [20]. We tested *mec-15(tm2691)* mutants in a behavioral assay that measures the rate of paralysis of animals in the presence of the acetylcholine esterase inhibitor aldicarb [22], [23]. When exposed to aldicarb, wild-type animals paralyze over a time course of 1–2 hours due to accumulation of acetylcholine in the extracellular fluid and subsequent permanent contraction of all body wall muscles. Changes in the rate of paralysis in this assay can result from changes in synaptic transmission in cholinergic or GABAergic motor neurons innervating body muscles. For example, mutant animals with reduced release of acetylcholine paralyze more slowly since extracellular accumulation of acetylcholine takes longer. Conversely, mutant animals with increased release of acetylcholine paralyze faster [24], [25]. Mutant animals with defects in GABA release also paralyze faster in the aldicarb assay since inhibition of muscle contraction by GABA signaling is reduced [26], [27]. We found that *mec-15* mutants paralyze faster than wild-type control animals (Figure 1A). To determine in which cells MEC-15 acts, we expressed *mec-15* specifically in GABAergic motor neurons of *mec-15* mutants using the promoter of the *unc-25* gene. Expression of this transgene in *mec-15* mutants completely rescued the fast paralysis, suggesting that MEC-15 acts in GABAergic motor neurons to affect GABAergic synaptic transmission (Figure 1A).

Next, we tested if *mec-15* is normally expressed in GABAergic motor neurons. We expressed GFP driven by the *mec-15* promoter (*Pmec-15*::GFP) in animals co-expressing the red fluorescent protein mCherry in GABAergic motor neurons with a cell-specific promoter (*Punc-25*::mCherry). Most, if not all, GABAergic motor neurons expressed *Pmec-15*::GFP (Figure 1B). In addition, P*mec-15*::GFP was expressed in many non-GABAergic motor neurons in the ventral nerve cord, indicating that *mec-15* is also expressed in cholinergic motor neurons (Figure 1B). P*mec-15*::GFP was also expressed in neurons in the head and tail as well as in other tissues as reported previously [20].

### GABA Release is Reduced in *mec-15* Mutants

**Figure 2:**
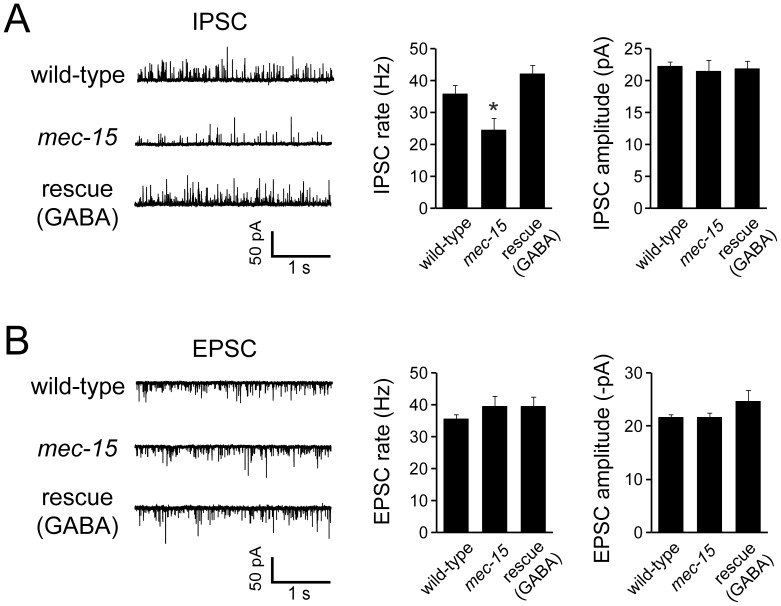
The rate of endogenous IPSCs, but not EPSCs, is reduced in the absence of MEC-15. (**A**) Recordings of endogenous inhibitory postsynaptic currents (IPSCs) were done on dissected adult *C. elegans*. IPSCs were recorded at 0 mV holding potential in the presence of 1 mM extracellular Ca^2+^. Left panels show representative traces recorded from wild-type animals, *mec-15* mutants and from *mec-15* mutants expressing *mec-15* in GABAergic neurons (rescue). Right panels show mean endogenous IPSC rates and amplitudes +/− SEM (n = 20, 13, and 12 for wild-type, *mec-15* and rescue, respectively). *indicates p  = 0.002, Student’s t-test. (**B**) Acetylcholine release is not changed in *mec-15* mutants. Endogenous excitatory postsynaptic currents (EPSCs) were measured as in (A) but at −60 mV holding potential. Left panels show representative traces, and right panels show mean endogenous EPSC rates and amplitudes +/− SEM of adult wild-type (n = 15), *mec-15* mutants (n = 12), and *mec-15* mutants expressing *mec-15* in GABAergic neurons (rescue) (n = 6).

To confirm a specific function of MEC-15 in GABAergic neurons, we measured synaptic activity at the NMJ in dissected animals by patch-clamp recordings from body wall muscles. In these dissected animals, basal nervous system activity drives endogenous excitatory and inhibitory postsynaptic currents [28]. *mec-15* mutants had a significantly lower endogenous IPSC rate than wild-type animals (Figure 2A). Importantly, this defect is due to a role of MEC-15 in GABAergic motor neurons, since it could be rescued by expressing *mec-15* specifically in these neurons using the *unc-25* promoter (Figure 2A). The amplitude of endogenous IPSCs was not affected (Figure 2A). Together, these results suggest that release of GABA is reduced in *mec-15* mutants while the GABA content of synaptic vesicles and muscle responsiveness to GABA are normal. In contrast, both the rate and amplitude of endogenous excitatory postsynaptic currents (EPSCs) were similar in wild-type and *mec-15* mutants (Figure 2B). These results are consistent with the results from the aldicarb assay and suggest that MEC-15 controls GABA release in GABAergic motor neurons, but does not affect cholinergic synaptic transmission.

### Changes in the Abundance of the Synaptic Vesicle Protein SNB-1 (Synaptobrevin) at GABAergic Synapses and in Cell Bodies in the Absence of MEC-15

**Figure 3:**
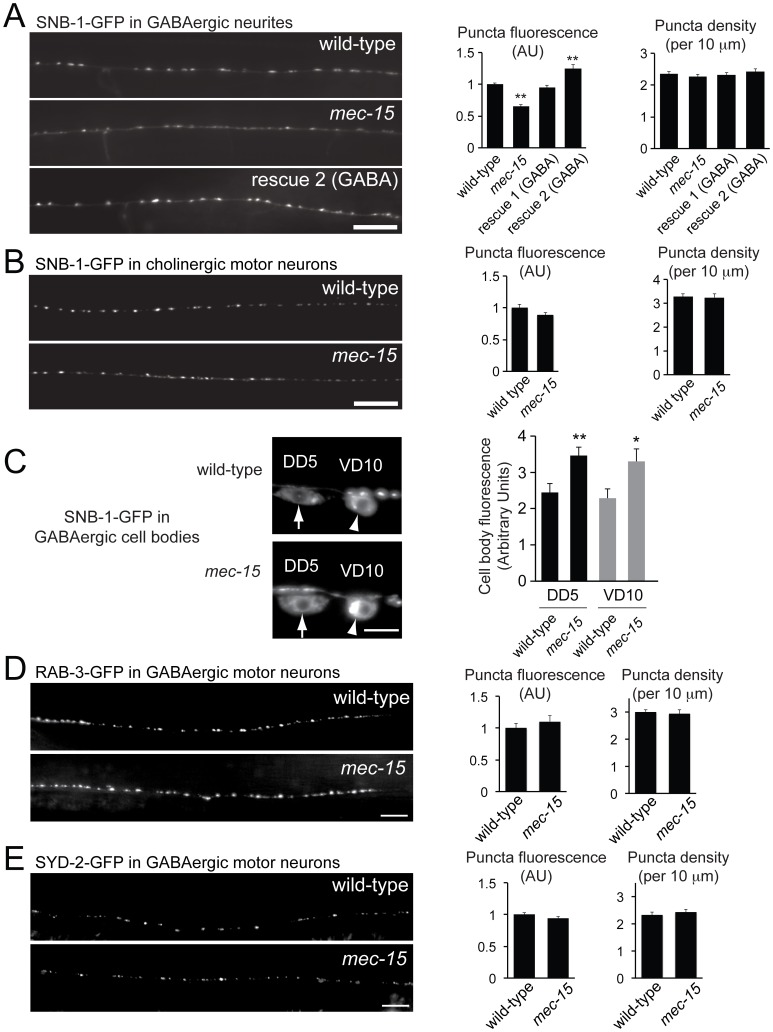
SNB-1-GFP is reduced at GABAergic synapses and accumulates in cell bodies in *mec-15* mutants. (**A**) Representative fluorescence images of SNB-1-GFP in neurites of GABAergic motor neurons in the posterior dorsal nerve cord from wild-type animals and *mec-15* mutants. *mec-15* mutants have reduced fluorescence intensity of SNB-1-GFP puncta compared to wild-type animals. Expression of *mec-15* in GABAergic neurons rescues this phenotype (rescue 2). Right panels show quantifications of puncta fluorescence and densities. Reduced puncta fluorescence of *mec-15* mutants is rescued by expressing *mec-15* in GABAergic motor neurons (two rescuing lines are shown). (**B**) Representative fluorescence images (left) and summary data (right) are shown for GFP-tagged SNB-1 in posterior dorsal cord axons of cholinergic motor neurons (expressed with the *unc-129* promoter) in wild-type and mec-15 animals. Right panels show quantifications. No significant differences were observed. (**C**) Representative fluorescence images of the GABAergic cell bodies DD5 and VD10 from wild-type animals and *mec-15* mutants expressing the synaptic vesicle protein SNB-1-GFP in GABAergic motor neurons (arrows and arrowheads point to DD5 and VD10, respectively). The right panels show quantifications. *mec-15* mutants have increased fluorescence intensity of SNB-1-GFP in cell bodies compared to wild-type animals. (**D**) Representative fluorescence images of RAB-3-GFP in neurites of GABAergic motor neurons in the posterior dorsal nerve cord from wild-type animals and *mec-15* mutants. Right panels show quantifications of puncta fluorescence and densities. Puncta fluorescence and synapse density are normal in *mec-15* mutants. (**E**) As in (D) except that GFP-tagged SYD-2 (liprin-α) was expressed. Neither puncta fluorescence nor synapse density is changed in *mec-15* mutants. All data are means +/− SEM from 20–30 images (A, B, D, E) or 15–20 cell bodies (C). **p<0.001, *p<0.01, Student’s t-test. Scale bars  = 10 µm (A, B, D, E) and 5 µm (C).

Neurotransmitter release is controlled by a variety of mechanisms. To begin to elucidate how MEC-15 affects synaptic transmission at GABAergic synapses, we determined the abundance of the GFP-tagged synaptic vesicle protein synaptobrevin (SNB-1-GFP) stably expressed in GABAergic motor neurons [29]. In wild-type animals, SNB-1-GFP localizes to punctate structures in the dorsal nerve cord that correspond to presynaptic sites in GABAergic motor neurons [30]. In *mec-15* mutants, the density of presynaptic puncta was not affected, but the fluorescence intensity of SNB-1-GFP at presynaptic sites was significantly reduced (Figure 3A). SNB-1-GFP is also diffusely localized between the punctate structures. This pool of SNB-1 is in the plasma membrane of neurites as part of the synaptic vesicle cycle [31]. The diffuse neurite fluorescence of SNB-1-GFP was also significantly reduced in *mec-15* mutants compared to wild-type animals (1+/−0.024, n = 26 and 0.74+/−0.027, n = 34, for wild-type and *mec-15* mutants, respectively, p<0.001). The reduced fluorescence intensity of SNB-1-GFP at punctate structures could be rescued to wild-type levels by expressing *mec-15* specifically in GABAergic motor neurons, indicating that MEC-15 functions in these neurons to control SNB-1-GFP abundance at synapses (Figure 3A, rescue 1). We also analyzed a second rescue line (rescue 2) that showed a significant increase of SNB-1-GFP fluorescence compared to wild-type, possibly due to overexpression of MEC-15 in this line.

We also analyzed if the synaptic abundance of SNB-1-GFP in cholinergic motor neurons was affected in *mec-15* mutants using transgenic animals expressing SNB-1-GFP specifically in cholinergic motor neurons under a promoter fragment of the *unc-129* gene [32]. In contrast to the reduced synaptic abundance of SNB-1-GFP in GABAergic neurons, we found that neither SNB-1-GFP fluorescence nor presynaptic density was affected in cholinergic motor neurons (Figure 3B), consistent with the results from the aldicarb experiments and electrophysiological recordings.

Changes in the abundance of SNB-1-GFP at synapses could result, among other things, from changes in protein expression, trafficking or degradation of SNB-1. Decreased trafficking could result in accumulation of SNB-1-GFP in GABAergic cell bodies. Therefore, we measured fluorescence intensity of SNB-1-GFP in the cell bodies of two GABAergic neurons, DD5 and VD10. In *mec-15* mutants, SNB-1-GFP abundance was significantly increased in these cells bodies (Figure 3C), consistent with the idea that MEC-15 controls the trafficking of SNB-1 between cell bodies and synapses.

To determine if the distribution of other synaptic proteins was affected in *mec-15* mutants, we analyzed the synaptic vesicle protein RAB-3 and the active zone protein SYD-2 (liprin-α) in GABAergic neurons [33], [34]. Fluorescently-tagged RAB-3, SYD-2 and SNB-1 co-localize at punctate structures that correspond to presynaptic sites [33]. The fluorescence intensity of punctate structures of RAB-3-GFP and SYD-2-GFP was not changed in *mec-15* mutants, and there was no change in the densities of these punctate structures (Figure 3D, E). Together, these data suggest that overall formation of GABAergic synapses occurs normally in *mec-15* mutants, and that MEC-15 specifically affects the distribution of SNB-1.

## Discussion

We analyzed the evolutionarily conserved and predicted SCF ubiquitin ligase subunit MEC-15 (FBXW9) in *C. elegans* to gain a more complete understanding of the role of specific ubiquitin ligases in the nervous system. Our results indicate that MEC-15 controls GABAergic synaptic transmission, possibly by regulating the abundance of the synaptic vesicle SNARE protein SNB-1 at synapses. Four lines of evidence support this. First, MEC-15 is expressed in GABAergic motor neurons in the ventral nerve cord. Second, *mec-15* mutants paralyze faster than wild-type animals in the aldicarb assay. This effect is rescued by expressing *mec-15* specifically in GABAergic motor neurons, suggesting that the fast paralysis is due to reduced GABA release in *mec-15* mutants. Third, electrophysiological recordings show a reduced endogenous IPSC rate in *mec-15* mutants, and this is rescued by expressing *mec-15* in GABAergic motor neurons. In addition, IPSC amplitude was not affected, indicating that signaling in postsynaptic muscle cells is not affected in *mec-15* mutants. Fourth, the abundance of the synaptic vesicle protein SNB-1 at GABAergic synapses, but not the density of synapses, is significantly reduced in *mec-15* mutants. Since the density of SNB-1-GFP punctate structures is not altered in the absence of *mec-15*, the decreased IPSC rate of *mec-15* mutants is unlikely caused by reduced synapse numbers, but instead could be due to reduced synaptic abundance of SNB-1. Reduced SNB-1 abundance could, in principle, result from defects in synapse assembly. This is not very likely since the density and abundance of two other presynaptic proteins, RAB-3 and SYD-2, is not changed in *mec-15* mutants. Instead, we found that SNB-1-GFP accumulates in cell bodies in *mec-15* mutants. Together, this suggests that MEC-15 affects the abundance of SNB-1 at synapses by controlling trafficking of SNB-1 between cell bodies and synapses; for example, export of SNB-1 from cell bodies could be reduced in the absence of MEC-15. The normal synaptic abundance of another synaptic vesicle protein, RAB-3, could hint that, unexpectedly, trafficking of SNB-1 and RAB-3 might be achieved by distinct mechanisms.

In contrast to the function of MEC-15 in GABAergic neurons, we did not detect a role for MEC-15 in cholinergic motor neurons. The fast paralysis in the aldicarb essay was efficiently rescued by expressing *mec-15* in GABAergic neurons indicating that potential changes in cholinergic motor neurons do not significantly contribute to this phenotype. Furthermore, endogenous EPSC rate and amplitude as well as abundance of SNB-1-GFP at cholinergic synapses are unchanged in *mec-15* mutants. Since MEC-15 is expressed in cholinergic neurons, it may have a function in these neurons that is not revealed in our experiments.

*mec-15* mutants were previously found to have diverse phenotypes in touch receptor neurons, including defects in synapse formation, enlarged cell bodies and reduced touch sensitivity [20], [21]. Touch receptor neurons in *mec-15* mutants had reduced numbers of synapses in the ventral nerve cord, reduced accumulation of GFP-tagged RAB-3 in the nerve ring, and enlarged cell bodies accumulated more GFP-tagged RAB-3 [20]. In contrast, synapse densities in GABAergic and cholinergic motor neurons are not changed in *mec-15* mutants and fluorescence intensity of RAB-3-GFP is similar to wild-type animals. Furthermore, defects of touch receptor neurons in the absence of MEC-15 are modulated by mutations in two tubulin genes that function specifically in these neurons [20]. Together, this could indicate that the function of MEC-15 in touch receptor neurons is distinct from the function in GABAergic and cholinergic motor neurons. For example, MEC-15 could have different ubiquitination targets in these three types of neurons. Alternatively, it is possible that the distinct phenotypes of *mec-15* mutants in these neurons are the result of the same underlying function of MEC-15 but differences in redundant or compensatory mechanisms. Since chemical synapses in touch receptor neurons are not required for the touch response, the reduced touch sensitivity in *mec-15* mutants did not allow conclusions about functional changes of chemical synapses [20], [35]. Here, we provide behavioral and electrophysiological evidence that MEC-15 promotes synaptic transmission in GABAergic motorneurons.

MEC-15 interacts with SKR-1 (Skp1), a common subunit of SCF ubiquitin ligases, in a yeast two-hybrid assay, supporting a function as part of an SCF ubiquitin ligase [36]. Definitive evidence will require biochemical analysis of MEC-15 and identification of ubiquitination targets. This will also be crucial to further define how MEC-15 functions in the nervous system. Since MEC-15 (FBXW9) is one of a few evolutionarily conserved F-box proteins, it should be informative to explore the function of FBXW9 in neurons of other species.

## Materials and Methods

### Strains, Mutants, Transgenes and *mec-15* Constructs

Animals were maintained at 20°C and fed OP50 *E. coli* as described [37]. The wild-type reference strain was N2 Bristol. Other strains and transgenes used in this study (strain information can be found at http://www.wormbase.org): *mec-15(tm2691)* was kindly provided by Shohei Mitani and outcrossed four times, *yuEx30* (P*mec-15*::GFP), *yuEx24* (P*unc-25p::mec-15*), *juIs1* (P*unc-25*::*SNB-1*-GFP) [29], *hpIs3* (P*unc-25*::GFP-*syd-2*) [34], *wyIs202* (P*flp-13*::GFP-*rab-3* and P*flp-13::*mCherry) [33], *nuIs152* (P*unc-129::snb-1-*GFP) [32].

The expression pattern of *mec-15* was determined using a 750 bp fragment from the start ATG of the *mec-15* gene to the 3′ end of the upstream gene as a promoter. This promoter was fused to GFP by PCR and directly injected into transgenic animals stably expressing mCherry in GABAergic neurons driven by the *unc-25* promoter (*yuIs10*) as described [38]. To rescue the *mec-15* mutant phenotypes, *mec-15* was amplified by PCR from cDNA and ligated into the *unc-25* promoter construct pSC325 (pIY73). Sequences of these constructs and PCR primers can be obtained upon request. Transgenic strains were generated by injecting N2 or *mec-15(tm2691)* animals with expression constructs (10–25 ng/µl) and the co-injection markers P*ttx-3*::GFP (40 ng/µl) or pKP1368 (P*myo-2*::NLS-DsRed, 5 ng/µl). Microinjections were performed using standard techniques as previously described [39].

### Aldicarb Assay

For analysis of sensitivity to the inhibitor of acetylcholinesterase aldicarb, paralysis of young adult worms was scored every 10 min, starting at 30 min, using 1 mM aldicarb (Chem Services), as previously described [40]. For each experiment, 20 worms per genotype were placed on NGM plates supplemented with aldicarb. Genotypes were blind to the scorer, and the analysis was repeated at least three times.

### Fluorescence Microscopy

Fluorescence microscopy experiments were performed as previously described [41]. Briefly, animals were immobilized with levamisole (10 mg/ml; Sigma) before imaging. Image stacks of young adult animals were acquired and maximum intensity projections generated using Metamorph software (Molecular Devices). Images of synapses were taken from the dorsal cord near the posterior gonad bend with the dorsal cord oriented towards the objective. Linescans of dorsal cord fluorescence were generated with Metamorph and analyzed in Igor Pro (WaveMetrics) using custom-written software to determine the fluorescence intensity, density and width of presynaptic puncta [31], [41]. Images of GABAergic cell bodies DD5 and VD10 were acquired in a similar way, but with the ventral side of animals oriented towards the objective. Cell body fluorescence was determined using Metamorph by measuring fluorescence from cell bodies and a directly adjacent area as background. In all experiments, images of animals with different genotypes were acquired in parallel. Images of 20–30 animals were used for quantification. Values reported in the figures are means ± SEM. Statistical significance was determined using the Student’s t test.

### Electrophysiology

Electrophysiology was done on dissected adults as described [42], and recording conditions were as described previously [43]. Briefly, worms were superfused in an extracellular solution containing 127 mM NaCl, 5 mM KCl, 26 mM NaHCO_3_, 1.25 mM NaH_2_PO_4_, 20 mM glucose, 3 mM CaCl_2_, and 3 mM MgCl_2_ (330 mOsm at pH 7.2), bubbled with 5% CO_2_ and 95% O_2_ at 20°C. For endogenous acetylcholine EPSCs, whole-cell patch-clamp recordings from body wall muscles were carried out at −60 mV using an internal solution containing 105 mM CH_3_O_3_SCs, 10 mM CsCl, 15 mM CsF, 4 mM MgCl_2_, 5 mM EGTA, 0.25 mM CaCl_2_, 10 mM HEPES, and 4 mM Na_2_ATP (315 mOsm, adjusted to pH 7.2 using CsOH). For endogenous GABA IPSCs, whole-cell recordings were carried out at 0 mV. Statistical significance was determined using Student’s t test.
